# Exploring the Impact of Chelating Agents on Copper Oxide Layer Formation and Morphology

**DOI:** 10.1021/acs.inorgchem.5c00068

**Published:** 2025-04-09

**Authors:** Damian Giziński, Anna Brudzisz, Jinhee Lee, Ruturaj Harishchandre, Jinsub Choi, Wojciech J. Stȩpniowski, Kirk J. Ziegler

**Affiliations:** † Faculty of Advanced Technologies and Chemistry, Military University of Technology, 00908 Warsaw, Poland; ‡ Department of Chemistry and Chemical Engineering, Inha University, Incheon 22212, Republic of Korea; § Department of Chemical Engineering, University of Florida, Gainesville, Florida 32611, United States; ⊥ Institute of Chemical Research of Catalonia (ICIQ), The Barcelona Institute of Science and Technology, 43007 Tarragona, Spain

## Abstract

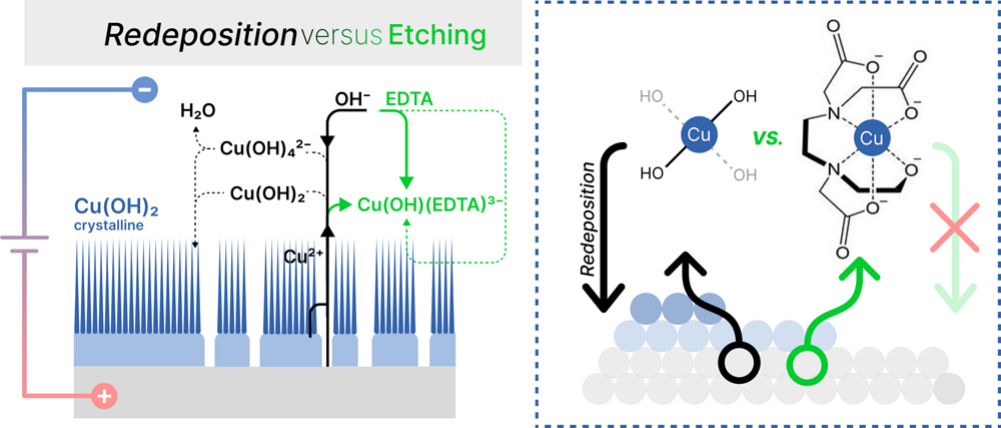

The morphological evolution of copper oxide surfaces during anodization was investigated using scanning electron microscopy (SEM), chronoamperometry, X-ray diffraction (XRD), and X-ray photoelectron spectroscopy (XPS). The addition of ethylenediaminetetraacetic acid (EDTA) as a Cu^2+^ chelating agent near the anode slows key mechanistic steps, providing insight into the factors driving the formation of crystalline Cu(OH)_2_ nanoneedles. The correlation of chronoamperometric data with SEM images revealed a four-stage process, beginning with the formation of an initial passive oxide layer, followed by nucleation. The comprehensive analysis of experimental results demonstrates that while electrochemical processes are necessary to initiate nanoneedle growth, the subsequent growth mechanism is not driven by direct electrochemical oxidation. Instead, supersaturation of the dissolved copper species near the electrode surface leads to nucleation and growth of Cu(OH)_2_ nanoneedles. The interaction with EDTA at different concentrations results in various morphologies of copper oxide surfaces, ranging from nanoneedles to disordered porous structures. This unique mechanism of copper oxide formation during anodization enables precise control of the surface properties, offering potential applications in catalysis and various energy technologies, including both production and storage.

## Abstract 2

The unique mechanism of copper oxide formation during anodization enables precise control of the surface properties, offering potential applications in catalysis and various energy technologies, including both production and storage.

## Introduction

The electrochemical oxidation of metals offers a wide array of opportunities to create nanostructured oxides. Anodic aluminum oxide (AAO) is particularly notable among these materials, distinguished by its hexagonally arranged, porous honeycomb morphology. This unique structure has significantly stimulated research interest in the field of metal electrochemical oxidation.^1,2^ Following the development of AAO, researchers have successfully applied electrochemical oxidation to various other metals, including Ti,^3^ Ta,^4^ Zr,^5^ Sn,^6^ W,^7^ Nb,^8^ and even stainless steel.^9^ These materials typically share characteristics such as fixed stoichiometry, amorphous structure, and nanoporous or nanotubular morphology. The resulting nanostructures have demonstrated significant value across a broad spectrum of applications. Beyond their crucial role in corrosion protection,^10,11^ these materials have found utility in diverse fields, including photocatalysis,^12^ nanofabrication,^13−15^ biomaterial development,^13^ drug delivery systems,^16^ photonics,^17^ and sensing technologies.^18^

The electrochemical oxidation of copper is particularly unique in the fundamental aspects of its formation and holds promise for breakthroughs in various emerging applications. During this process, copper forms a range of compounds, including Cu_2_O, CuO, and Cu(OH)_2_. Unlike the amorphous nature of anodic oxides typically formed from other metals, anodized copper materials exhibit crystalline structures. This crystallinity is evident in copper anodization studies using 0.1 M NH_4_HCO_3_ solutions, which produce crystalline forms such as Cu_2_O, CuO, Cu_4_O_3_, and Cu_2_(OH)_2_CO_3_.^19^ Another intriguing feature of copper electrochemical oxidation is the diverse morphology of its products (nanoparticles, nanowires, nanopores), which distinguishes it from the anodization of other metals. For instance, anodizing copper in 0.1 M NH_4_HCO_3_ results in nanowires clustered in pom-pom-like structures,^19^ while using alkaline electrolytes, such as 1.0 M KOH, produces nanoneedles.^20,21^ This morphological versatility adds to the unique characteristics of electrochemically oxidized copper.

Understanding the mechanistic aspects of copper electrochemical oxidation is crucial for tailoring materials to meet the demands of key applications. These applications span a wide range, including the electrochemical reduction of carbon dioxide,^22,23^ the photocatalytic decomposition of pollutants,^24,25^ photoelectrochemical reactions such as hydrogen generation,^26^ and the assembly of direct methanol fuel cells.^20,27^ The unique morphology and crystalline nature of copper anodization products set this process apart from the electrochemical oxidation of other metals. Although conventional anodizing typically involves electrochemical growth and field-assisted etching, the copper oxidation mechanism must include additional factors to account for the diverse structures observed. Understanding this intricate mechanism is crucial for controlling the chemical composition, crystallinity, and morphology of the resulting nanostructures, thereby optimizing their performance in various technological applications.

The process by which copper oxide forms during electrochemical oxidation remains a topic of discussion among researchers. The current literature proposes two potential pathways: direct electrochemical oxidation and redeposition of dissolved species. In the electrochemical oxidation pathway, metallic Cu is initially oxidized to copper(I) species like Cu(OH)_2_^–^, CuOH, and Cu_2_O, with Cu(I) species potentially further oxidizing to Cu(OH)_2_ or CuO.^28^ Alternatively, the mechanism of redeposition suggests that in alkaline environments, water-insoluble compounds, such as CuO and Cu(OH)_2_, dissolve as water-soluble Cu(OH)_4_^2–^ species.^28^ These soluble species could be subsequently redeposited on the oxidizing Cu surface, forming the oxide layer. The dominant process and its correlation with the diverse morphology of the observed surface structures remain unclear. Resolving this uncertainty is crucial to understanding the unique characteristics of copper electrochemical oxidation products and controlling the composition and morphology of the resulting nanostructures.

Although our previous study explored the role of ethylenediaminetetraacetic acid (EDTA) in the electrochemical oxidation of copper,^29^ the current work represents a fundamental study that controls kinetics and mass transport to elucidate the mechanisms occurring at the electrode–electrolyte interface. Using lower potentials and a strongly alkaline environment, we slow the reactions, enabling a detailed examination of the initial steps in oxide growth. The highly alkaline solution increases the effectiveness of EDTA in chelating copper ions, providing unprecedented clarity in the observation of the redeposition process. In contrast to the limited morphological changes observed in our earlier work, this study unveils a diverse array of nanostructures, offering critical insights into their formation mechanisms. Through meticulous control of electrochemical conditions, we establish direct correlations between inhibition of Cu(OH)_2_ redeposition and specific alterations in the chemical composition and morphology of the oxidation products. This innovative approach not only illuminates the pivotal role of redeposition in determining oxide/hydroxide layer characteristics but also provides a robust and versatile framework for comprehending and manipulating copper electrochemical oxidation across various conditions. Our findings lay the groundwork for the precise tailoring of copper oxide nanostructures for a wide range of applications.

## Experimental Methods

### Reagents

Ultrapure high-resistance water (15 Ω·cm) was used in all experiments. Sodium hydroxide (98.8%) was obtained from POCh Basics (Poland). Disodium ethylenediaminetetraacetic acid dihydrate (Na_2_H_2_(EDTA)·2H_2_O, analytical grade) and H_3_PO_4_ (85%) were purchased from Chempur (Poland). Hydrochloric acid (HCl, 35–38%, analytical grade) was sourced from POCh. Copper foil (99.99%) was purchased from Avanti (Poland). Before use, each copper foil was electropolished in a 10 M H_3_PO_4_ solution for 3 min at 10 V. After electropolishing, 2 × 2 cm copper foil coupons were thoroughly cleaned with deionized water and ethanol.

### Methods

Before electrochemical anodization, each copper coupon was kept in diluted HCl for 5 min to remove the native oxide layer. All electrochemical methods were performed in a one-compartment cell equipped with a three-electrode assembly using a PalmSens4 potentiostat (PalmSens BV, Netherlands). Platinum and Hg/HgO/3 M KOH were used as counter electrode and reference electrode, respectively. The copper electrode was mounted at the bottom of the cell in a horizontal position. The area of the copper electrode was limited to 1.78 cm^2^ with a rubber O-ring.

**Figure 1:**
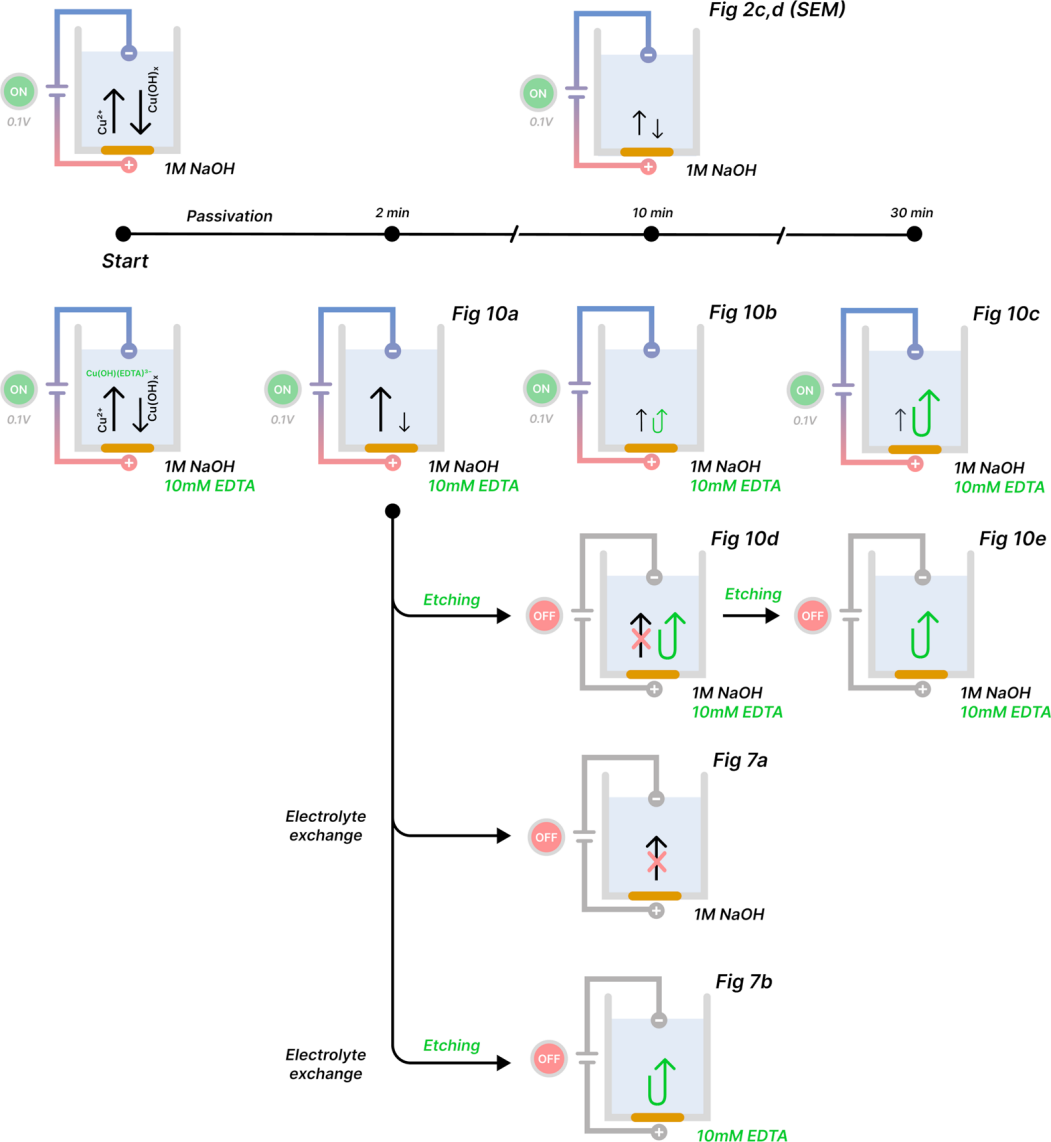
Schematic representation of the experimental workflow. The designed experiments aim to elucidate the conflicting effects of EDTA (illustrated with green arrows) and applied cell voltage (illustrated with black arrows) on the formation of the anodic layer on copper. The numerical labels correspond to specific figures with SEM micrographs presented in this study.

Potentiostatic anodization was performed for 10 min in chronoamperometric mode at a constant cell potential of 0.1 V. Each experiment used 75 mL of electrolyte, which consisted of 1 M NaOH supplemented with varying concentrations of Na_2_H_2_(EDTA), ranging from 1 to 500 mM. Following anodization, the copper foils were thoroughly rinsed with deionized water and immediately dried using an Ar gas stream. Additionally, a series of control experiments were conducted to systematically evaluate the influence of electrolyte composition, applied current, and time on the formation of the anodic layer on copper. A schematic representation of these control experiments is illustrated in Figure 1.

### Characterization

Cyclic voltammetry scans were recorded for copper in 1 M NaOH with the addition of Na_2_H_2_(EDTA)·2H_2_O at concentrations of 1, 10, and 100 mM. For reference, a scan in pure 1 M NaOH solution was also measured. Measurements were done at 50 mV/s to ensure a noticeable separation of the two main anodic peaks. The deconvolution of the overlapping peaks was performed with the PSTrace software (version 5.9.4515, PalmSens BV) using the semidifferentiation method.^30^ The anodic sweep of each voltammogram with two overlapping peaks was processed with a set fractional derivative order of 0.01 for the reversible peak model.

X-ray diffraction (XRD) analysis was used for the identification of the crystalline phase and was performed with an Ultima IV diffractometer (Rigaku Co., Japan) using a Co Kα source. The diffraction was measured in the angle range 2θ of 10°–100° with a step size of 0.02° and an acquisition rate of 1°/min. The interpretation of diffractograms and phase identification was done using Match! software (version 3.11, Crystal Impact).

Scanning electron microscopy (SEM) was performed on a Quanta 3D field emission SE microscope (FEI, USA). Planar samples were immobilized on the sample holder in the microscope chamber with conductive carbon tape. The morphology analysis of the oxidized materials was performed in low vacuum mode at an accelerating voltage of 10 kV.

X-ray photoelectron spectroscopy (XPS) was applied for the investigation of the chemical composition and valence states of surface species on the modified copper electrodes. XPS measurements were performed with the NEXSA surface analysis system (ThermoFisher Scientific, USA) equipped with monochromated Al Kα source (1486.68 eV). All generated XPS data was converted to VAMAS format and further processed with CasaXPS software. XPS spectra were deconvoluted to identify the chemical states for both the Cu 2p and O 1s regions. The Cu 2p_3/2_ peaks were observed at 932.4, 933.6, and 934.7 eV, corresponding to Cu_2_O, CuO, and Cu(OH)_2_, respectively. Additionally, the O 1s spectrum exhibited peaks at 529.8 (oxide), 531.3 (O–H species), and 532.8 eV (O (S)).

## Results

Several electrochemical reactions can occur at the electrode during Cu anodization. Table 1 lists the reactions expected at the anode in alkaline media for different potentials. The initial release of Cu^+^ from the surface occurs because it is the least energy intensive step in copper oxidation. Although Cu^+^ is not a stable species, it readily reacts with OH^–^ to form Cu_2_O on the surface through half reaction (HR1). At higher potentials, Cu(II) species begin to form. As seen in these half-reactions, the pH of the solution affects the electrochemistry.^31^ The alkaline conditions of the electrolyte result in the formation of several species of copper hydroxide during anodization. The half-reactions at the anode dictate which species are introduced into the electrolyte. For example, half reaction (HR2) introduces Cu(OH)_4_^2–^ into the electrolyte near the anode. These species introduced into the electrolyte then interact with other aqueous species. Some of the primary reactions involving copper species in the electrolyte are shown in Table 2. Therefore, the concentration of species in the electrolyte can influence the species at the surface, either by directly altering the formation of the oxide layer or by introducing reactions that alter the morphology of the oxide layer.

**Table 1:** Electrochemical Half-Reactions at the Copper Surface during Anodization in Alkaline Electrolytes

#	half reaction	*E*_*i*_^o^ (V)^a^
HR1	2Cu_(s)_ + 2OH^–^ → Cu_2_O_(s)_ + H_2_O + 2e^–^	0.4331^28^
HR2	Cu_(s)_ + 4OH^–^ → Cu(OH)_4_^2–^ + 2e^–^	0.6506^28^
HR3	Cu_2_O_(s)_ + 2OH^–^ + H_2_O → 2Cu(OH)_2(s)_ + 2e^–^	0.6989^28^
HR4	Cu_2_O_(s)_ + 6OH^–^ + H_2_O → 2Cu(OH)_4_^2–^ + 2e^–^	0.7304^28^
HR5	Cu_(s)_ + 2OH^–^→CuO_(s)_ + H_2_O + 2e^–^	0.7848^32^

a Standard electrode potentials are referenced to a reversible hydrogen electrode.

**Table 2:** Primary Equilibrium Reactions for Copper Species in the Electrolyte and Their Known Logarithmic Constants at 25 °C

#	equilibrium reaction	log *K*_*i*_	ref
**Cu(I) species**			
R1	2Cu^+^ + H_2_O ⇌ Cu_2_O_(s)_ + 2H^+^	1.62	(33)
R2	Cu^+^ + H_2_O ⇌ CuOH_(s)_ + H^+^	–0.91	(33)
R3	Cu_2_O_(s)_ + 2OH^–^ + H_2_O ⇌ 2Cu(OH)_2_^–^	–5.84	(28)
**Cu(I) disproportionation**			
R4	2Cu^+^ ⇌ Cu_(s)_ + Cu^2+^	6.08	a
**Cu(II) species**			
R5	Cu^2+^ + OH^–^ ⇌ Cu(OH)^+^	5.8	(33, 34)^b^
R6	Cu^2+^ + 2OH^–^ ⇌ Cu(OH)_2_^0^_(aq)_	10.5	(33, 34)^b^
R7	Cu^2+^ + 2OH^–^ ⇌ Cu(OH)_2 (s)_	19.15	(33, 34)^b^
R8	Cu^2+^ + 3OH^–^ ⇌ Cu(OH)_3_^–^	14.2	(33, 34)^b^
R8	Cu^2+^ + 4OH^–^ ⇌ Cu(OH)_4_^2–^	16.9	(34)^c^
R9	2Cu^2+^ + 2OH^–^ ⇌ Cu_2_(OH)_2_^2+^	17.4	(33, 34)^b^
R10	3Cu^2+^ + 4H_2_O ⇌ Cu_3_(OH)_4_^2+^ + 4H^+^	–20.76	(33)
R11	Cu^2+^ + H_2_O ⇌ CuO_(s)_ + 2H^+^	–7.95	(33)
**EDTA (ethylenediaminetetraacetic anion) species**			
R12	Cu^2+^ + EDTA^4–^ ⇌ Cu(EDTA)^2–^	20.5	(34)
R13	Cu^2+^ + OH^–^ + EDTA^4–^ ⇌ Cu(OH)(EDTA)^3–^	22.6	(34)

a Based on standard reduction potentials for cuprous and cupric and the Nernst equation.

b Combination of the reaction from ref (34) with water dissociation yields the reported value in ref (33).

c Large discrepancy in values but the value from ref (34) is self-consistent with reactions (R7) and (R9).

In this study, a known chelating agent, EDTA, is introduced to scavenge copper ions during the anodization of copper. As shown in reactions (R13) and (R14) in Table 2, the large log *K*_*i*_ indicates that EDTA has a strong affinity for Cu^2+^. The results for anodization in pure alkaline solutions are presented first. Changes in the oxide layer are then discussed when a small amount of EDTA is added to the electrolyte.

### Electrochemical Oxidation of Copper in Alkaline Electrolytes

**Figure 2:**
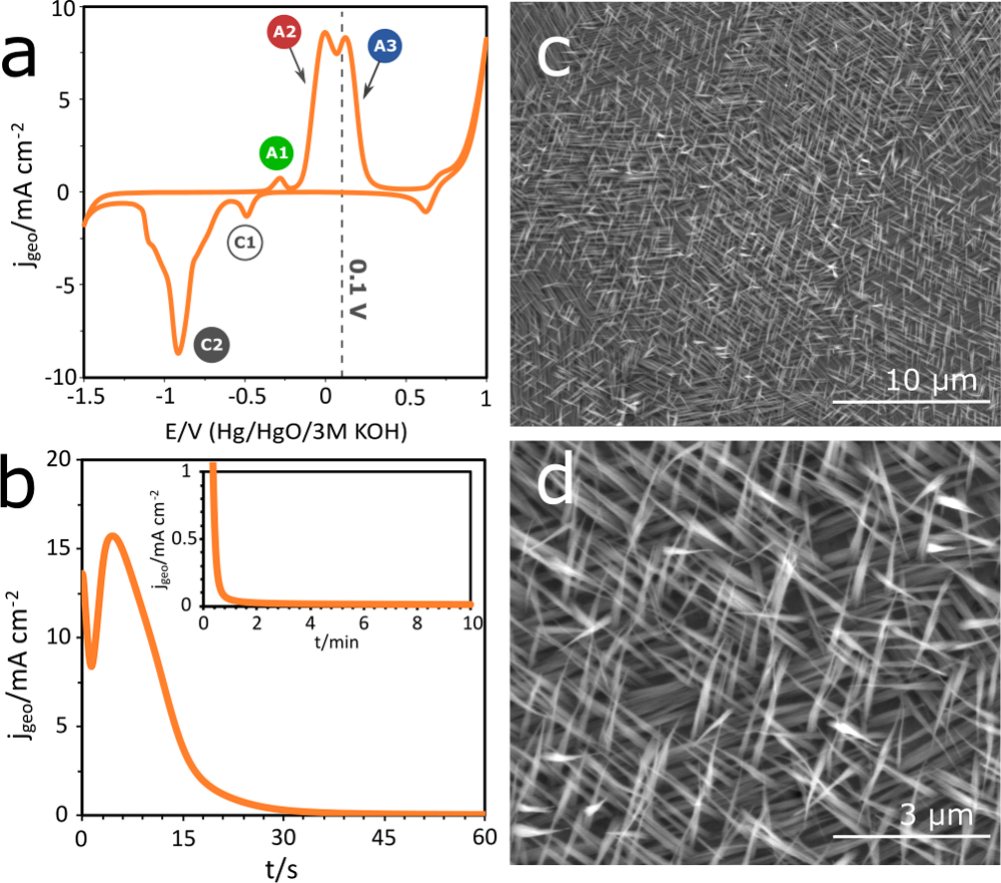
(a) Cyclic voltammogram and (b) current density vs time of pure copper anodized in 1 M NaOH solution. (c, d) SEM images of copper anodized for 10 min at 0.1 V (dashed line in the voltammogram).

The voltammogram for bulk copper in 1 M NaOH solution is depicted in Figure 2a. Its shape and peak positions are in agreement with previously reported voltammograms obtained for copper in alkaline media.^28,32,35^ Three anodic peaks were identified and marked as 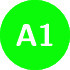, 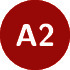, and 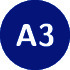. The first oxidation peak 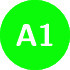 that appeared during the potential sweep from negative to positive values was relatively short and located at −0.29 V (Hg/HgO). It represents the initial copper oxidation that yields Cu_2_O,^35,36^ according to half reaction (HR1). Further scanning of the potential revealed a significantly more intense double peak. These peaks correspond to the formation of copper species at a higher oxidation state. Peak deconvolution separated the overlapping peaks into their representative peak positions of 0 and 0.13 V, respectively. The peak 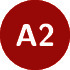 is assigned to the formation of copper(II) in hydroxide forms, as soluble Cu(OH)_4_^2–^ through half reactions (HR2) and (HR4) and insoluble Cu(OH)_2_ through half reaction (HR3).^32^ These species can result from oxidation of a Cu or Cu_2_O surface. At the highest potential, copper is oxidized to CuO and is represented by peak 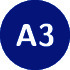 in Figure 2. The reverse cathodic sweep from positive to negative potentials showed two main reduction peaks labeled 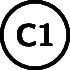 and 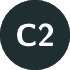. The short 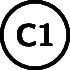 peak is located at −0.50 V and represents the reduction of CuO to Cu. The tall 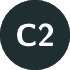 peak is located at −0.92 V and corresponds to the reduction of Cu(OH)_2_/Cu(OH)_4_^2–^ to metallic Cu.

Anodic reactions will affect the composition of the copper surface. Copper anodization was conducted at 0.1 V, which is the potential corresponding to the maximum peak of 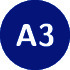. Therefore, the operating conditions were sufficient to trigger the complete sequence of anodic reactions described earlier. The transient current during potentiostatic anodization is shown in Figure 2b. Immediately after the process is initiated, the current drops as the metal surface becomes covered by an insulating oxide coating. After ∼1 s, the current starts to rise again as the initial oxide layer breaks down, leading to passivity loss (current increase). This suggests that the underlying metal is once again locally exposed to the electrolyte. Approximately 5 s into the process, the freshly exposed metal undergoes oxidation. As the anodization progresses, the current flow is gradually suppressed as an increasingly thicker oxide layer is formed. This shape of the chronoamperometric curve clearly implies a two-step mechanism for oxide layer formation. The system eventually attains a steady-state value of ∼15 μA cm^–2^ after 40 s. Because the oxide coating does not completely insulate the metal electrode, the oxide layer continues to grow slowly.

### Changes to Electrochemical Oxidation Associated with EDTA

As EDTA interacts with copper species both on the surface and in solution, it can affect the concentrations of various species on both the surface and in the electrolyte. These localized concentration changes can lead to changes in the chemistry, morphology, and composition of the oxide layer formed. To investigate the influence of EDTA on the copper oxide layer in an alkaline electrolyte, a series of experiments was performed in 1 M NaOH supplemented with different concentrations of EDTA.

#### Electrochemical Reactions

**Figure 3:**
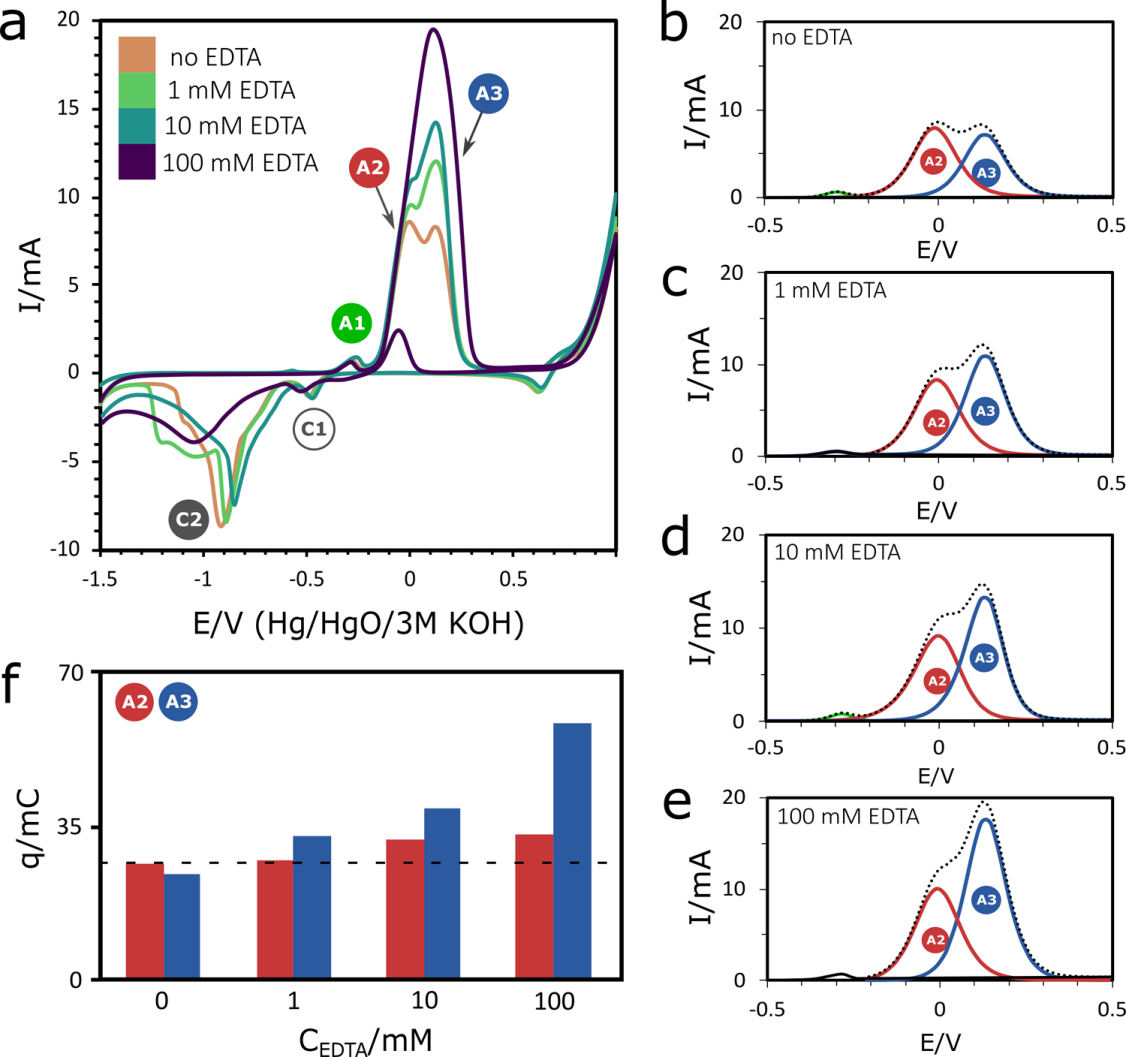
(a) Cyclic voltammograms of the Cu-EDTA system in 1 M NaOH and with the addition of EDTA in 1, 10, and 100 mM concentrations. The peaks 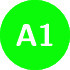, 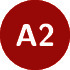, and 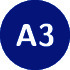 identify the anodic reactions, while 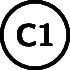 and 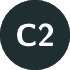 identify the cathodic reactions. (b–e) Deconvolution of the overlapping anodic peaks of the voltammograms is presented for EDTA concentrations of (b) 0, (c) 1, (d) 10, and (e) 100 mM. (f) Total charge is compared for the major peaks observed in the voltammograms.

Figure 3a compares the voltammograms in pure alkaline solutions with those containing a small concentration of EDTA. The shape of the curves is generally maintained at all concentrations. Those containing EDTA have a higher current and the relative importance of specific reactions is a function of the EDTA concentration. Interestingly, the reverse sweep at 100 mM EDTA shows a new anodic current. This new anodic peak suggests that the bare Cu surface was exposed to the electrolyte, allowing the anodic reactions to occur during the reverse sweep. In other words, a reaction in the electrolyte removes the oxide layer faster than the electrochemical reduction of the copper oxide.

The differences in the anodic current can be seen better after the voltammograms have been deconvoluted into their respective bands. The voltammogram in the alkaline electrolyte (no EDTA) showed that the maximum current of the 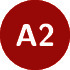 peak is higher than the one recorded for the consecutive 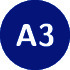 peak (Figure 3b). As shown in Figure 3c, this proportion changes when EDTA is introduced into the electrolyte. Interestingly, the 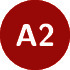 peak remains nearly identical to the pure alkaline curve with both exhibiting a peak current of ∼8 mA. However, 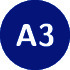 peak has increased significantly with even a small amount of EDTA. This increase in the 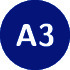 peak continues as the EDTA concentration increases further to 10 and 100 mM (Figure 3d,e).

The peak area represents the charge flow that accompanies an electrochemical process. Figure 3f summarizes the charge flow changes for various concentrations of EDTA. This plot shows that the 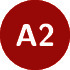 peak associated with the formation of Cu(OH)_4_^2–^ and Cu(OH)_2_ was not affected significantly by the presence of EDTA, while the 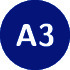 peak associated with the formation of CuO was enhanced. Similar behavior was also observed in the reverse sweep shown in Figure 3a. The 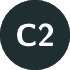 peak assigned to the reduction of the Cu(OH)_2_ species disappears when EDTA is at its highest concentration in the electrolyte, whereas the 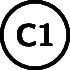 peak associated with the reduction of CuO is observed at all the EDTA concentrations tested.

**Figure 4:**
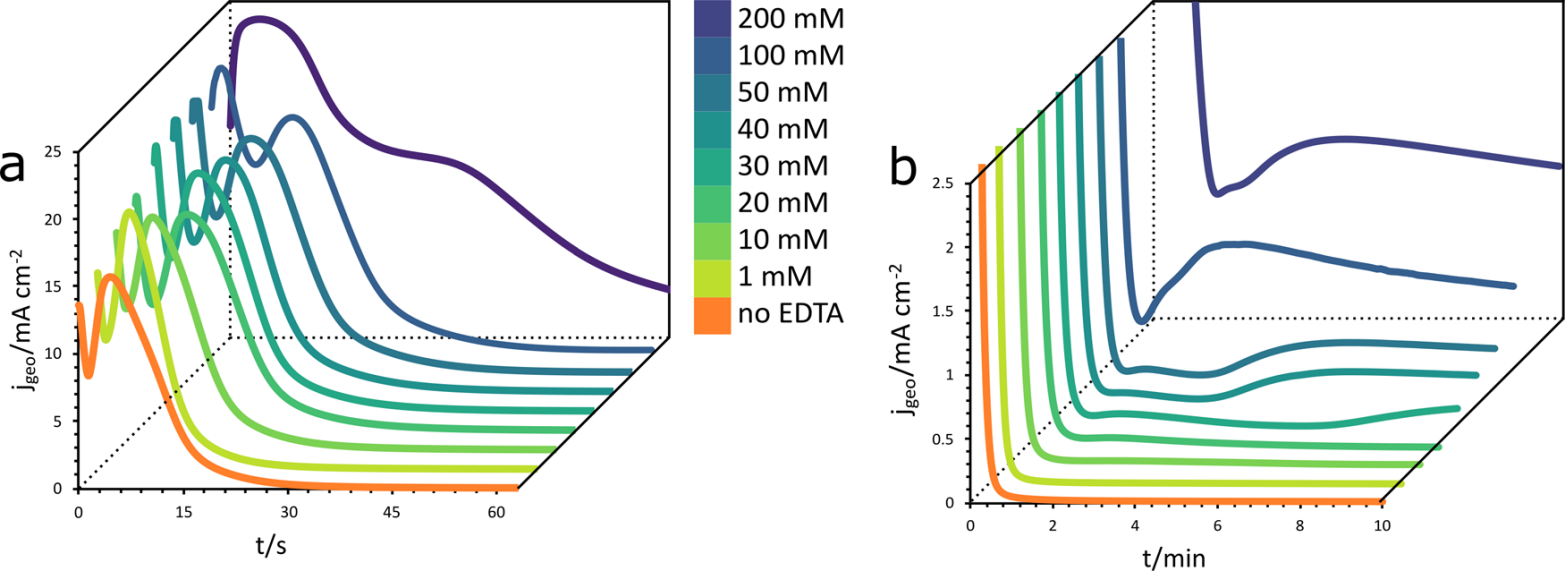
Current density vs time during the (a) first minute and (b) entire time range of copper anodization in alkaline solutions containing various concentrations of EDTA.

Figure 4 compares the transient current during anodization for various concentrations of EDTA at different time scales. At small time scales (Figure 4a), the general shape of the chronoamperometric curves remains the same until an EDTA concentration of 200 mM. Although the shape is the same, the peak associated with the secondary oxide layer (after loss of passivity) stretches to longer time scales as the concentration of EDTA increases. In addition, the depth of the initial drop in current is reduced. This behavior suggests that EDTA slows the growth of the initial oxide layer. At 200 mM, it appears that the initial formation of the oxide layer is completely suppressed.

At low concentrations of EDTA (≤20 mM), the current density reaches its minimal value after ∼1 min and it remains stable until the end of the passivation process, as shown in Figure 4b. However, this steady state is disrupted at EDTA concentrations ≥30 mM. For the 30 mM EDTA concentration, the current density starts to rise again after 8 min, indicating that the underlying metal is once again exposed to the electrolyte. Further increases in the EDTA concentration in the electrolyte result in a shorter steady-state period. At 50 mM of EDTA, the current starts to rise after 3 min where it reaches a new steady-state value of ∼0.37 mA cm^–2^. At EDTA concentrations ≥100 mM, the steady-state periods have disappeared entirely and at least two peaks are observed in the chronoamperometric curves (approximately 0.2 and 4 min). Note that this is also the concentration at which the electrolyte removed the oxide layer during the reverse CV sweep (Figure 3), resulting in a new anodic current during the reverse sweep.

#### Morphological Changes

**Figure 5:**
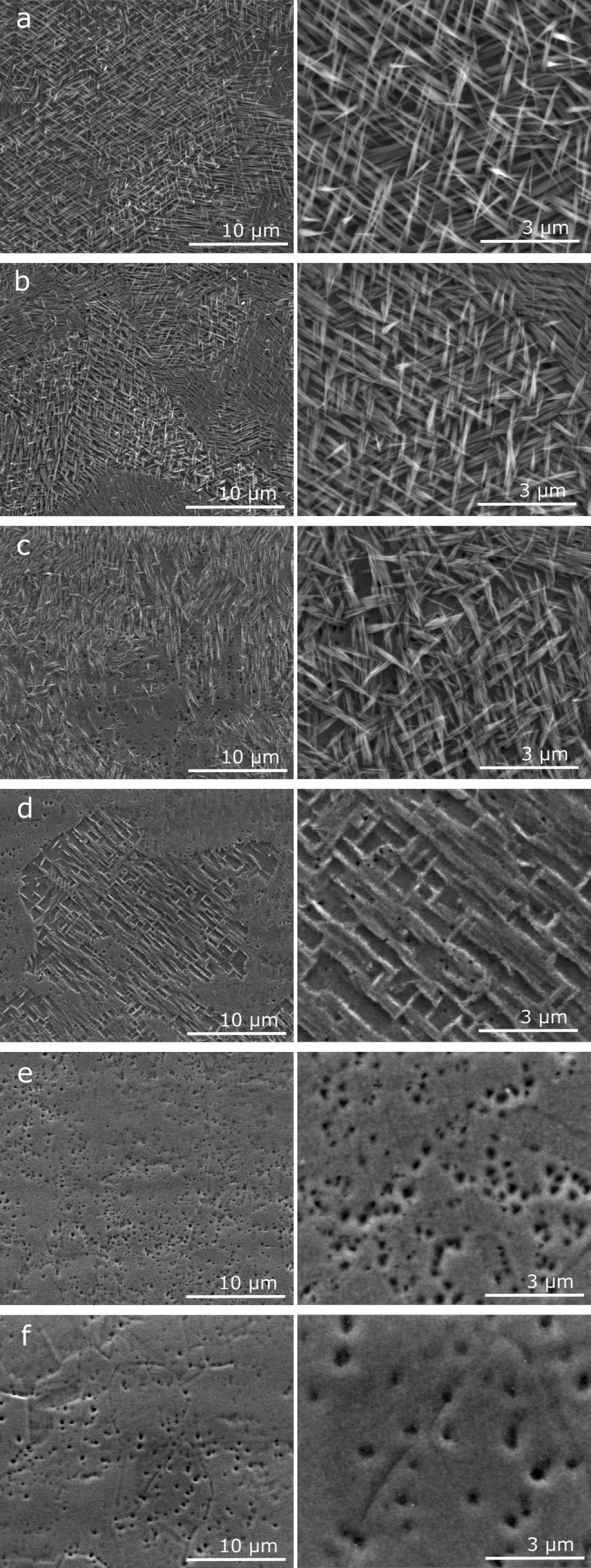
SEM images at two magnifications of the copper surface anodized in 1 M NaOH and EDTA concentrations of (a) 1, (b) 10, (c) 20, (d) 30, (e) 40, and (f) 100 mM.

SEM images of copper anodized at various concentrations of EDTA are shown in Figure 5. Comparison with the images for standard anodization shown in Figure 2c shows that the lowest concentration of EDTA in the electrolyte (1 mM) had a negligible effect on the formation of nanoneedles. However, Figure 5b–d (see Figure S1 for all concentrations) shows that copper anodization conducted with EDTA at concentrations of 20 mM or higher results in the formation of oxide layers with diverse surface morphologies. Interestingly, the surface morphology transitions from the nanoneedle assembly at low concentrations to a porous compact layer at higher concentrations. At 20 mM EDTA concentrations, the nanoneedles are not as well-defined and have a less ordered layout across the surface. The nanoneedles appear flattened and lie largely on the surface of the copper. For copper anodized in the presence of 30 mM EDTA (Figure 5c), the surface appears to only contain remnants of nanoneedles that once covered the surface or indicate that the nanoneedles mask the etching of the underlying oxide. These features are completely removed after 10 min of anodization in the electrolyte containing 40 mM EDTA. Above this concentration, only a compact layer with unevenly distributed pores was observed. As shown in Figure 5e, the oxide layer grown on the copper surface at 100 mM EDTA also shows a compact layer morphology pitted with unevenly distributed pores. However, these pores are visibly wider and have rounded edges. At the highest tested EDTA concentration of 500 mM (see Figure S1), the compact oxide layer appears to be very thin, with only small patches of oxide observed. Their shape resembles the grain distribution of the bare metal surface. During anodization with 500 mM EDTA, there was a deep blue color near the copper electrode, indicating a significant amount of copper dissolution.

**Figure 6:**
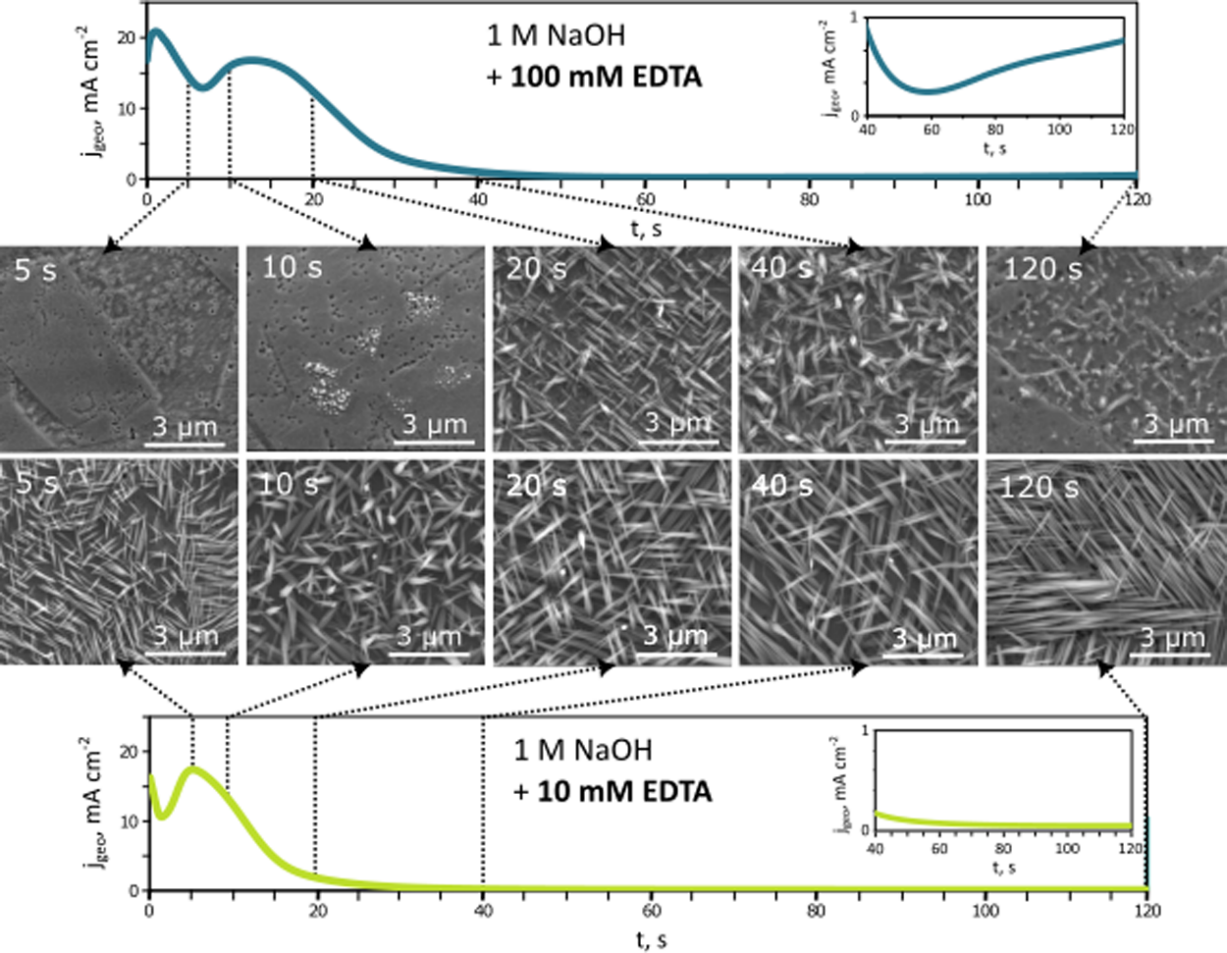
Time-dependent evolution of the surface morphology during copper anodization at 0.1 V in 1 M NaOH with 10 (top) and 100 mM EDTA (bottom). Specific SEM images are shown relative to the chronoamperometric curve measured during anodization.

To further investigate the formation of the oxide layer on copper in the presence of EDTA, the anodization treatment was arrested at specific times along the chronoamperometric curve for two EDTA concentrations of 10 and 100 mM, as shown in Figure 6. These arrested anodization experiments allowed changes in the morphology of the oxide coating to be observed within the initial phase of the process.

When anodized in 10 mM EDTA, the initial drop in current density represents the formation of a thin compact oxide layer on the surface. This layer breaks down as the anodization proceeds and pores are formed (see Figure S2), enabling further oxidation of copper. As a result, the current density rises to its maximum value after 5 s. This is enough time for the nanoneedles to form and cover the surface as a second tier of the oxide coating. This additional layer leads to the second drop in the current density that eventually attains a steady state. After 10 s, the nanoneedles are still oriented perpendicular to the metal surface. However, the morphology after 20 s already indicates that their vertical assembly is disrupted and the nanoneedles appear to lie flat on the surface. This behavior continues as time progresses. At completion, the nanoneedles lie completely flat on the surface. While the morphology changes, there are also changes in the size and shape of the nanoneedles. During the transient stage, the lengths of the nanoneedles do not change much. After a low steady-state current is attained (where most electrochemical reactions have ceased), the needles get longer. This evolution in morphology clearly shows a complex process that is driven by electrochemical oxidation and chemical reactions that alter the morphology while in the electrolyte.

For copper anodized in an electrolyte containing 100 mM of EDTA, more distinct changes in morphology were observed as the process evolved. The chronoamperometric curve shows two broad maxima. Therefore, the initial oxide layer formation was considerably slowed down, enabling two distinct stages of the oxide layer construction with well-defined current maxima to be observed. The first increase in current density is associated with the reorganization of the initial compact oxide layer that creates a thin protective coating on the metal surface after 5 s. The grain boundaries are still noticeable after this time, indicating the thinness of the compact layer. Subsequently, the compact layer breaks down, and the current density starts to rise again. At this concentration of EDTA, pores formed during this stage are more easily observed. Small particles start to appear on the surface after 10 s of anodization. These particles are likely the nuclei for the crystallization of nanoneedles. As these nanoneedles grow, the thick coating leads to the second drop in current density. The time it takes for the nanoneedles to develop is significantly longer (≳10 s) than in the low EDTA concentration case (≲5 s). Furthermore, their size and shape imply that the formation process is affected by the presence of EDTA. The nanoneedles reach their maximum length at the end of the transient period (∼40 s). At this stage, the response at high concentrations starts to deviate from the behavior observed at low concentrations of EDTA. As shown in the inset of the chronoamperometric curve in Figure 6, the current rises again after ∼60 s, indicating that a new process has disrupted the steady-state condition. As seen in the final SEM image (120 s), severe decomposition of the external nanostructured layer of the surface has occurred. The surface morphology has now flattened again, suggesting that the current rise is associated with a chemical process that breaks down the oxide layer.

**Figure 7:**
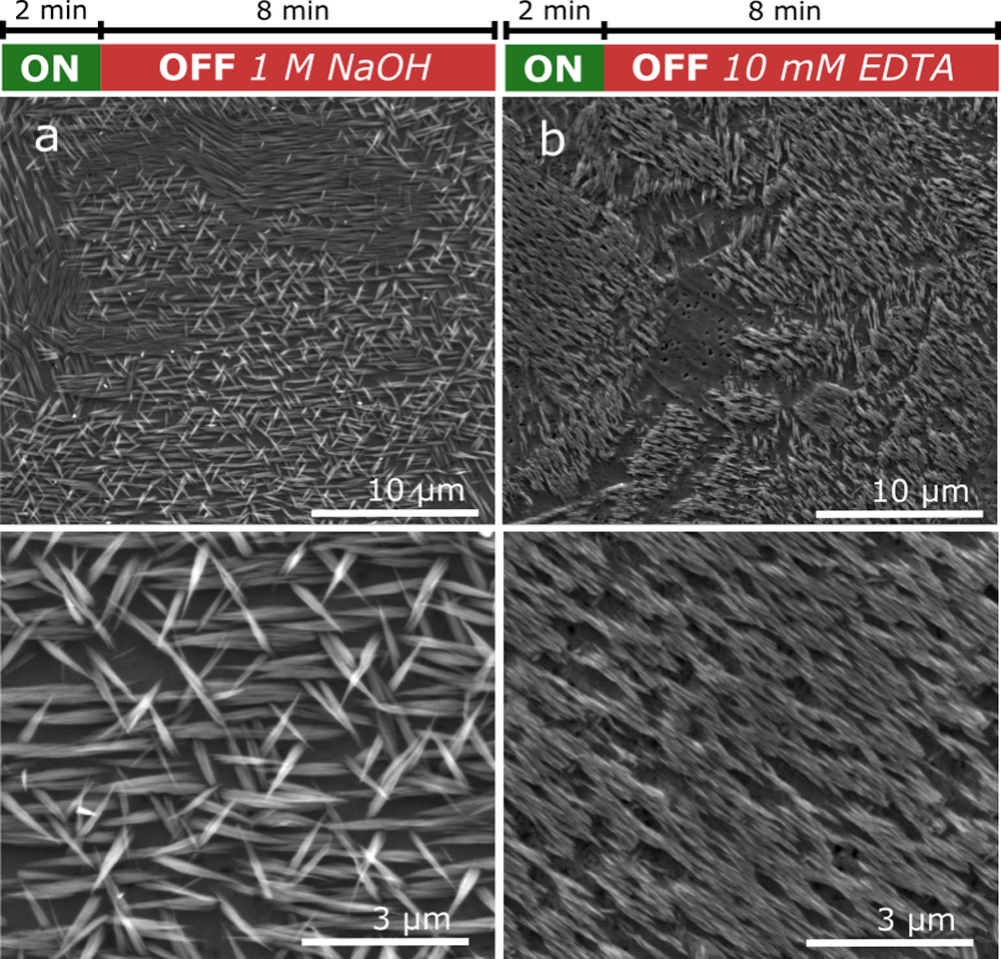
SEM images at two magnifications of the copper surface after anodization in 1 M NaOH with 10 mM EDTA that were washed and subsequently transferred to a solution of either pure (a) 1 M NaOH or (b) 10 mM EDTA. No external cell potential was applied after transfer. The time associated with each step of the process is shown at the top of the figure. The anodization time is shown in green, while the time remaining in the electrolyte after anodization is shown in red.

Although EDTA has a strong chelating affinity for Cu^2+^ (see Table 2), an additional experiment was performed to verify that EDTA is responsible for the etching behavior. Copper was anodized for 2 min in 10 mM EDTA. After this short anodization, the copper surfaces were washed and immersed into electrolyte containing either pure 1 M NaOH or pure 10 mM EDTA with no applied potential. As shown in Figure 7, the oxide layer was significantly degraded when exposed to the EDTA-containing solution. While the morphology of the nanoneedles was maintained, individual nanoneedles were much shorter and only observed in aligned patches. However, subjecting the copper surface to pure 1 M NaOH had little effect on the morphology of the nanoneedles.

#### Composition Changes

**Figure 8:**
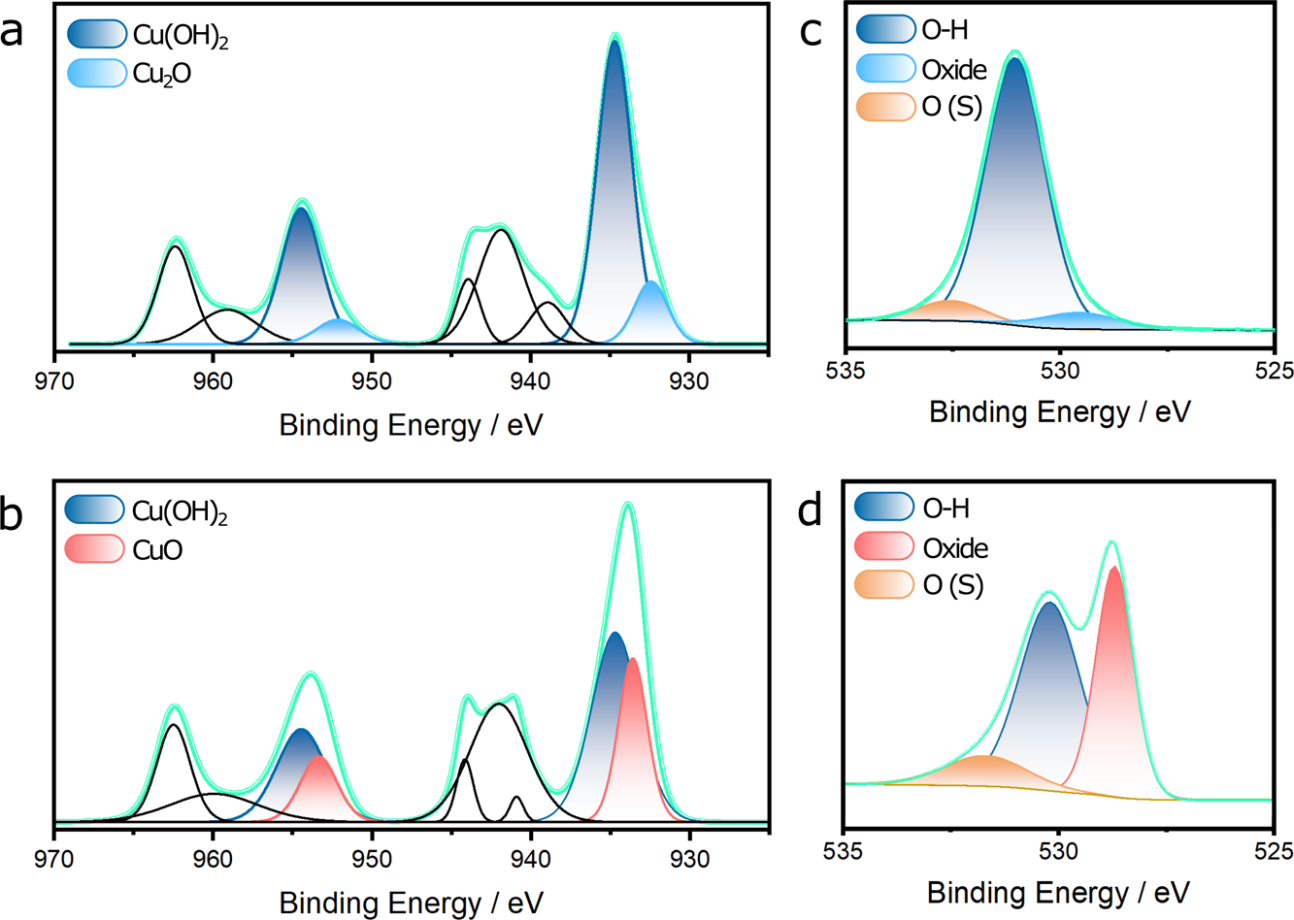
High-resolution XPS spectra of (a, b) Cu 2p and (c, d) O 1s regions for copper anodized in 1 M NaOH with EDTA at a concentration of (a, c) 10 and (b, d) 30 mM.

The chemical state of the oxide surface is shown in the XPS data of Figure 8 for two different concentrations of EDTA. Copper anodized in electrolyte containing 10 mM EDTA showed no significant differences in surface composition compared to those anodized without EDTA (Figure S8). Figure 8a shows that Cu(OH)_2_ dominates the surface composition at low concentrations of EDTA, with Cu_2_O also present in appreciable amounts (15 at %). The O 1s signal in Figure 8c further supports the presence of these two copper species. Specifically, the strongest oxygen response at ∼531 eV correlates to hydroxide species.^37,38^ Similar chemical compositions were observed in alkaline solutions elsewhere.^39^

The spectra of Cu anodized in 30 mM EDTA show distinct differences. The Cu 2p spectra, particularly the shape of the satellite peaks in the 2p_3/2_ region, reveal differences in the chemical states of copper between Figure 8a,b. These satellite peaks are characteristic features of Cu^2+^ species. The variations in their shape and intensity observed in Figure 8a,b are consistent with the reported spectral features of Cu(OH)_2_ and CuO, respectively, as described by Biesinger et al.^40^ Although the dominant copper species is still Cu(OH)_2_, CuO replaces Cu_2_O as the secondary constituent. To quantify this change, XPS peak deconvolution was performed in the Cu 2p_3/2_ region, analyzing the peaks corresponding to Cu(OH)_2_, Cu_2_O, and CuO (see Table S1). For the sample anodized in 30 mM EDTA, the peak area attributed to CuO was estimated to be 35 at %. Furthermore, analysis of the O 1s region in Figure 8d confirms this trend, showing a proportion of oxides (39 at %) similar to that determined from the Cu spectra, indicating that hydroxides contribute less to the surface composition. XPS spectra of copper anodized in 100 mM EDTA (Figure S3) appear nearly identical to these data, indicating that there are no significant changes in surface states at higher concentrations of EDTA.

**Figure 9:**
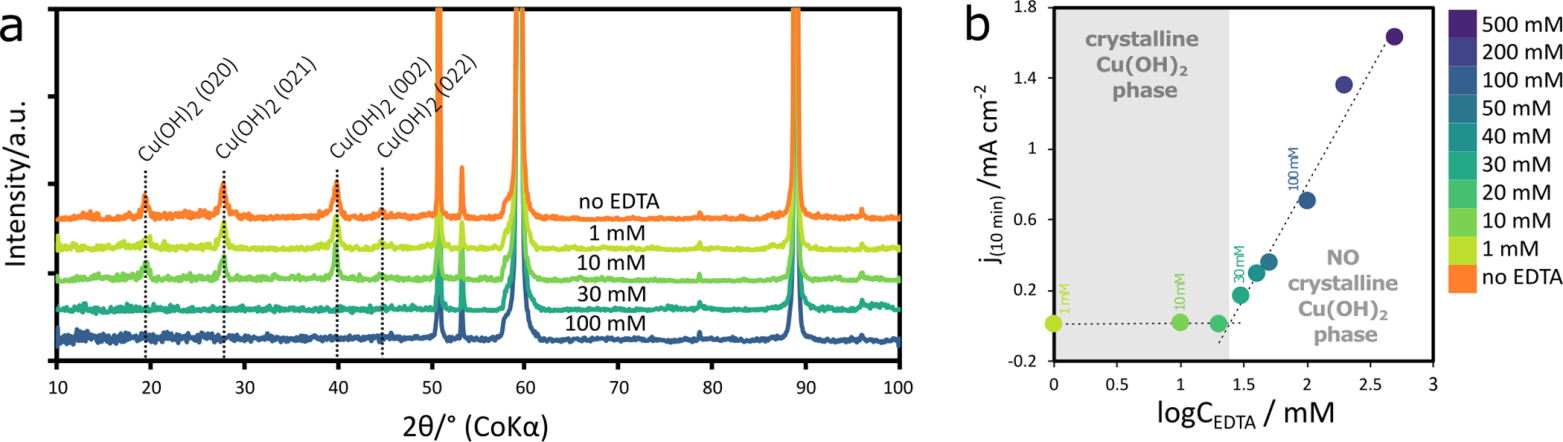
(a) XRD of copper anodized in a 1 M NaOH electrolyte with the addition of EDTA at various concentrations between 1 and 100 mM. Specific peaks associated with Cu(OH)_2_ are labeled. The unmarked peaks originate from the copper substrate. (b) Current density after 10 min of anodization versus logarithmic molar concentration of EDTA in the electrolyte.

The composition of the oxide layers formed at different concentrations of EDTA (0, 1, 10, 30, and 100 mM) was also analyzed by XRD. As shown in Figure 9a, the only crystalline phase detected in addition to the Cu metal was Cu(OH)_2_. However, Cu(OH)_2_ was only detected in oxide layers formed at low EDTA concentrations. The remaining surfaces (≳30 mM) had no detectable amount of Cu(OH)_2_. The lack of any crystalline material at higher concentrations of EDTA suggests that the oxide layer is either amorphous in nature or too thin to be detected.

The SEM images shown in Figures 5 and S1 show that nanoneedles are only observed when anodized in solutions containing less than 30 mM of EDTA. At higher concentrations, only the compact layer was observed. XRD and XPS analysis clearly show that the nanoneedles formed on the surface consist of a crystalline Cu(OH)_2_ phase. Although Cu(OH)_2_ is observed in the XPS spectra at higher concentrations of EDTA, it likely consists of a thin surface layer, since it is generally accepted that the penetration depth of XPS is ∼10 nm.^41^ Therefore, the final oxide coatings grown in electrolytes with low EDTA content indicate the formation of a two-tier structure consisting of crystalline Cu(OH)_2_ nanoneedles on top of a compact layer of amorphous copper oxide.

Figure 9b shows the logarithmic concentration of EDTA in the electrolyte versus the current density recorded at the end of the anodization (i.e., 10 min). At low concentrations, the current density is minimal as a result of the intact oxide layer. However, the final current increases logarithmically with EDTA concentration once a threshold concentration is reached. This transition in current density is also closely correlated with the presence of crystalline Cu(OH)_2_ nanoneedles, indicating that the increase in current is associated with the exposure of the underlying metal surface to the electrolyte. Therefore, the threshold value can estimate the boundary condition to maintain a layer of Cu(OH)_2_ nanoneedles on the surface, which requires EDTA concentrations below 25 mM.

## Discussion

The results demonstrate that multiple species are present at the anode during copper anodization. CV, XPS, and XRD data indicate that Cu_2_O, Cu(OH)_2_, and CuO are located at the anode at various stages of the process. As Tables 1 and 2 show, these species can be generated by electrochemical reactions or redeposited from the solution phase. The XRD data in Figure 9 indicate that the nanoneedles are the only crystalline material formed in appreciable quantities and are composed of Cu(OH)_2_.

It is apparent from Figure 6 that the transient period (before a steady state current is established) is responsible for the initiation of the nanostructured morphology. At low EDTA concentrations of 10 mM, the EDTA does not have an appreciable effect on nanoneedle growth, since the final structures obtained after 120 s look similar to the structures formed in the absence of EDTA (see Figure 2). After the transient period ends at ∼40 s, the nanoneedles continue to grow longer during the steady-state low current period.

**Figure 10:**
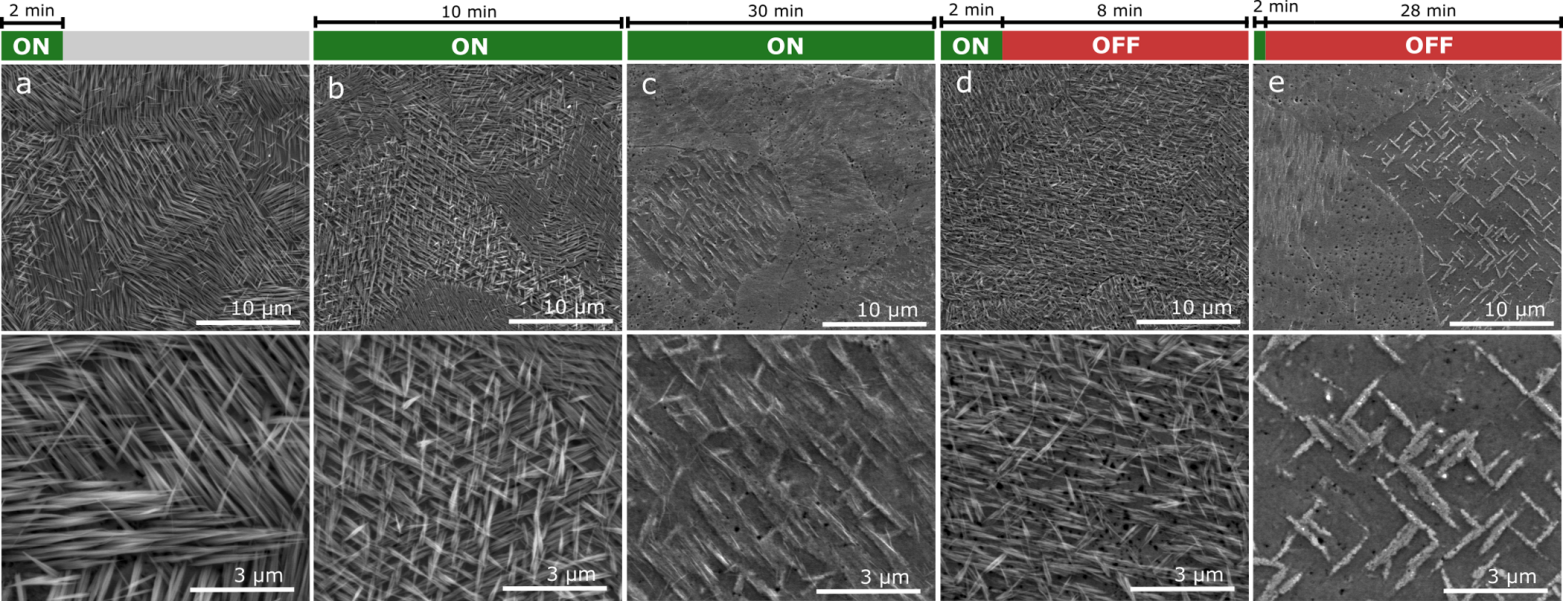
SEM images at two magnifications of the surface morphology of copper for different process times in 1 M NaOH with a 10 mM EDTA electrolyte. The time associated with each step of the process is shown at the top of the figure. The anodization time is shown in green, while the time remaining in the electrolyte after anodization is shown in red.

If Cu(OH)_2_ were formed by an electrochemical process, it would be expected that longer anodization times would lead to longer nanoneedles. However, the images in Figure 10a–c show that longer anodization times at 10 mM EDTA do not lead to longer nanoneedles. The images show that the nanoneedles are longest at 2 min and continue to get shorter with longer anodization times. After 30 min, virtually no nanoneedles remain. These results would suggest that the electric current does not have an effect on the nanostructures. This conclusion is supported by the CV data in Figure 3f, which shows that the peak associated with the production of Cu(OH)_2_ from electrochemical processes is not significantly altered by EDTA. However, if the bias is applied for only 2 min and remain in the EDTA solution for extended periods, as shown in Figure 10d,e, then there are fewer nanoneedles and they are noticeably shorter in length, indicating that the current is at least important to maintain these structures.

### Proposed Mechanism for Nanoneedle Formation

**Figure 11:**
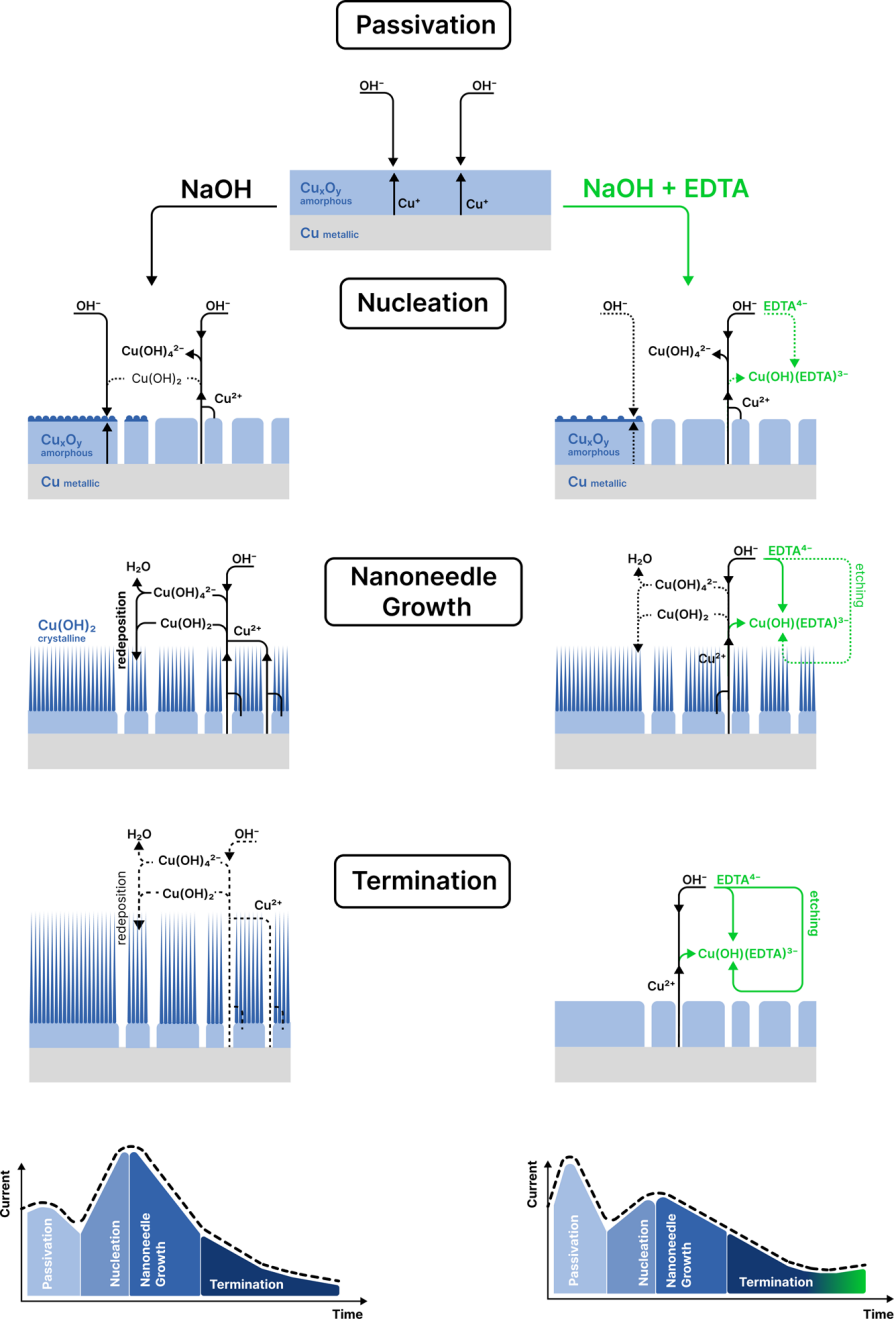
Schematic representation of the four stages of nanoneedle growth during copper anodization, which consist of passivation, nucleation, nanoneedle growth, and termination. Chemical reactions and structures formed at the surface during anodization in alkaline media are shown in the absence (left) and presence of EDTA (right). Processes that are just being initiated or inhibited are shown as dashed lines. Processes involving EDTA are shown in green.

The comprehensive analysis of the surface morphology, electrochemical behavior, and oxide composition of the experimental results helps to identify the underlying principles that guide the formation of crystalline nanoneedles of Cu(OH)_2_. Based on these results, a four-stage mechanism is proposed in Figure 11 that encompasses the interplay of chemical and physical phenomena that drive the formation and growth of the oxide layer on copper surfaces in the absence and presence of EDTA.

#### Passivation

The shape of the chronoamperometric curves for copper anodization in alkaline electrolytes shows two current maxima that appear consecutively after applying an external potential (Figure 4). This implies a two-step mechanism for the formation of an oxide layer in copper.^20,21^ As shown in Figure 11, copper is first oxidized to form a passive layer resulting in the first drop in current seen in Figures 4 and 6. Analysis of the time-dependent evolution of the surface morphology in Figure 6 revealed that this first step is associated with the coverage of the copper surface with a thin compact barrier layer.^42^ The XPS data in Figure 8a,c suggest that this initial layer is Cu_2_O formed by half reaction (HR1). However, the XPS data in Figure 8b,d indicate that CuO generated from half reaction (HR5) becomes dominant at higher concentrations of EDTA, indicating that both Cu_2_O and CuO form but that Cu_2_O is being consumed. This is also supported by the CV data in Figure 3 that shows the dominant formation of the CuO (peak 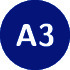). The XRD data in Figure 9 shows only crystalline Cu(OH)_2_, therefore, the oxide layer consisting of both Cu_2_O and CuO is amorphous.

#### Nucleation

Within a few seconds, the thin oxide layer breaks, allowing further oxidative restructuring of the surface, as shown in Figure 11. This initial Cu_*x*_O_*y*_ layer starts to break down and form pores, as observed in the SEM images of Figures 6 and S2. This process is similar to the anodization of aluminum, which shows similar chronoamperometric curves at this stage and significant pore formation.^43,44^ The current in Figures 4 and 6 starts to rise again after the formation of pores due to increased exposure of the underlying metal surface to the electrolyte. These pores create channels for further oxidation of copper, leading to the migration of Cu^2+^ ions from the metal surface^43,44^ and thus increases the current. Under high pH conditions, soluble Cu(OH)_4_^2–^and insoluble Cu(OH)_2_ are expected to form,^35,45^ according to reactions (R9) and (R7), respectively.

As shown in Figure 11, the addition of the EDTA chelating agent to the electrolyte creates an alternative route for the consumption of the Cu^2+^ ions released during electrochemical oxidation. The dissolved copper can be chelated with EDTA to form a stable Cu(OH)(EDTA)^3–^ coordination complex,^34,46^ according to reaction (R14). Therefore, the addition of EDTA elongates the various stages of the process, allowing one to accurately investigate the complex nature of this process.

The time-dependent evolution data in Figure 6 (at 100 mM EDTA) shows that no nanoneedles are formed prior to this stage. At this point, the electrolyte near the electrode is saturated with the Cu^2+^ ions that are produced. When the concentration reaches supersaturation, the formation of nuclei is triggered. The data in Figure 6 suggest that nuclei form on the surface during this stage of rising current and pore formation.

#### Nanoneedle Growth

As shown by the analysis of the evolution of the surface morphology in Figure 6, nanoneedles begin to develop after pores are formed in the oxide layer. The XRD data in Figure 9 indicate that the nanoneedles are composed of Cu(OH)_2_. Typically, the current would reach a steady state after pore formation, as observed with aluminum anodization.^47^ However, it is observed that the current falls rather quickly after the formation of pores in the anodization of copper, as shown in Figures 4 and 6. This drop in current is typically associated with increased resistance or reduced transport to complete the electrochemical reactions. The resistance may increase as the oxide layer thickens, or it could be that the formation of nanoneedles on the surface hinders the transport of OH^–^ to the surface.

As shown in Figure 11, consumption of Cu^2+^ ions by EDTA inhibits the growth of nanoneedles by lowering the local concentration of Cu^2+^ near the electrode. However, even though inhibited, nanoneedle formation occurs at even high concentrations of EDTA, as seen in Figure 6. The data in Figure 10 illustrate the importance of Cu^2+^ ions to the formation of nanoneedles. A current is needed to continue to supply Cu^2+^ ions that feed the growth of the nanoneedles. Without this small current and Cu^2+^ supply, EDTA simply shortens the nanoneedles through an etching process. This shows that the formation of Cu(OH)_2_ nanoneedles includes the utilization of Cu(II) ions released from the solid state during electrochemical oxidation.

#### Termination

As anodization continues, the current drops until it finally reaches a steady state (∼30 s). As the oxide layer and the nanoneedle array coat the metal surface with an increasingly dense layer, the release of Cu^2+^ ions into the solution is significantly reduced, as shown in Figure 11. Once the rate of Cu^2+^ loss due to redeposition is greater than the production of Cu^2+^ from anodization, the nanoneedles cease to grow.

After reaching steady-state conditions (≳30 s), the etching effect of EDTA becomes more relevant. This shifts the equilibrium of the system in favor of removing the oxides, including the Cu(OH)_2_ nanoneedles and the amorphous Cu_2_O and CuO. Exposure to high concentrations of EDTA or long periods of time demonstrates a wide variety of surface morphologies seen in Figures 5 and S1. As these structures are attacked by EDTA, a new anodic current occurs at long times in Figures 4 and 6, indicating that the surface becomes exposed to new reactions.

## Conclusions

EDTA is a strong chelating agent for Cu^2+^ ions that not only functions as an effective etching agent for copper oxide species, but also alters the equilibrium of numerous reactions, enabling the investigation of various chemical processes during copper anodization. The concentration of EDTA in the electrolyte significantly influences the morphology, chemical composition, and crystallinity of the formed layer. While EDTA induces gradual dissolution of the nanoneedles, these nanostructures remain observable at EDTA concentrations below ∼25 mM. At higher concentrations, a diverse array of morphological structures emerges, progressing from a plateau-like structure to a disordered, porous surface morphology, and ultimately to a thin passive layer.

The correlation of results from multiple experiments elucidated the mechanism of electrochemical copper oxidation and highlighted the crucial role of Cu^2+^ ions ejected from the pores of the surface oxide layers, which could be modulated by EDTA. The growth process begins with the formation of amorphous copper oxide, followed by the ejection of Cu^2+^ ions that lead to the nucleation and growth of Cu(OH)_2_ nanoneedles via redeposition from the electrolyte. This mechanism, unique to copper and distinct from other metals, is fundamental to controlling the surface of copper oxides. By manipulating Cu(II) hydroxide redeposition through precise addition of EDTA, we can fine-tune the morphology, composition, and crystallinity of the nanostructures. This mechanistic insight enables tailored customization of the copper oxide surface for various applications, including the electrochemical reduction of CO_2_ into hydrocarbons or alcohols, which could address challenges related to global warming.

## Supporting Information Available

SEM images of morphological changes, XPS spectra, and Auger spectra at various concentrations of EDTA (PDF)
