# Supplementary material for: Exploring the Impact of Chelating Agents on Copper Oxide Layer Formation and Morphology
Source: Inorg Chem. 2025 Apr 9;64(15):7437–49. doi: 10.1021/acs.inorgchem.5c00068 (PMC12015832; doi:10.1021/acs.inorgchem.5c00068)
Supplement: Supplementary file 1 — ic5c00068_si_001.pdf [file ic5c00068_si_001.pdf]

Exploring the Impact of Chelating Agents on Copper Oxide Layer Formation and Morphology

Damian Giziński,[†] Anna Brudzisz,[†] Jinhee Lee,[‡] Ruturaj Harishchandre,[¶] Jinsub Choi,[‡] Wojciech J. Stępniowski,[*,†] and Kirk J. Ziegler[*,†,¶]

†*Faculty of Advanced Technologies and Chemistry, Military University of Technology, 00908 Warsaw, Poland*

‡*Department of Chemistry and Chemical Engineering, Inha University, Incheon 22212, Republic of Korea*

¶*Department of Chemical Engineering, University of Florida, Gainesville, Florida 32611, USA*

E-mail: wojciech.stepniowski@wat.edu.pl; kziegler@che.ufl.edu

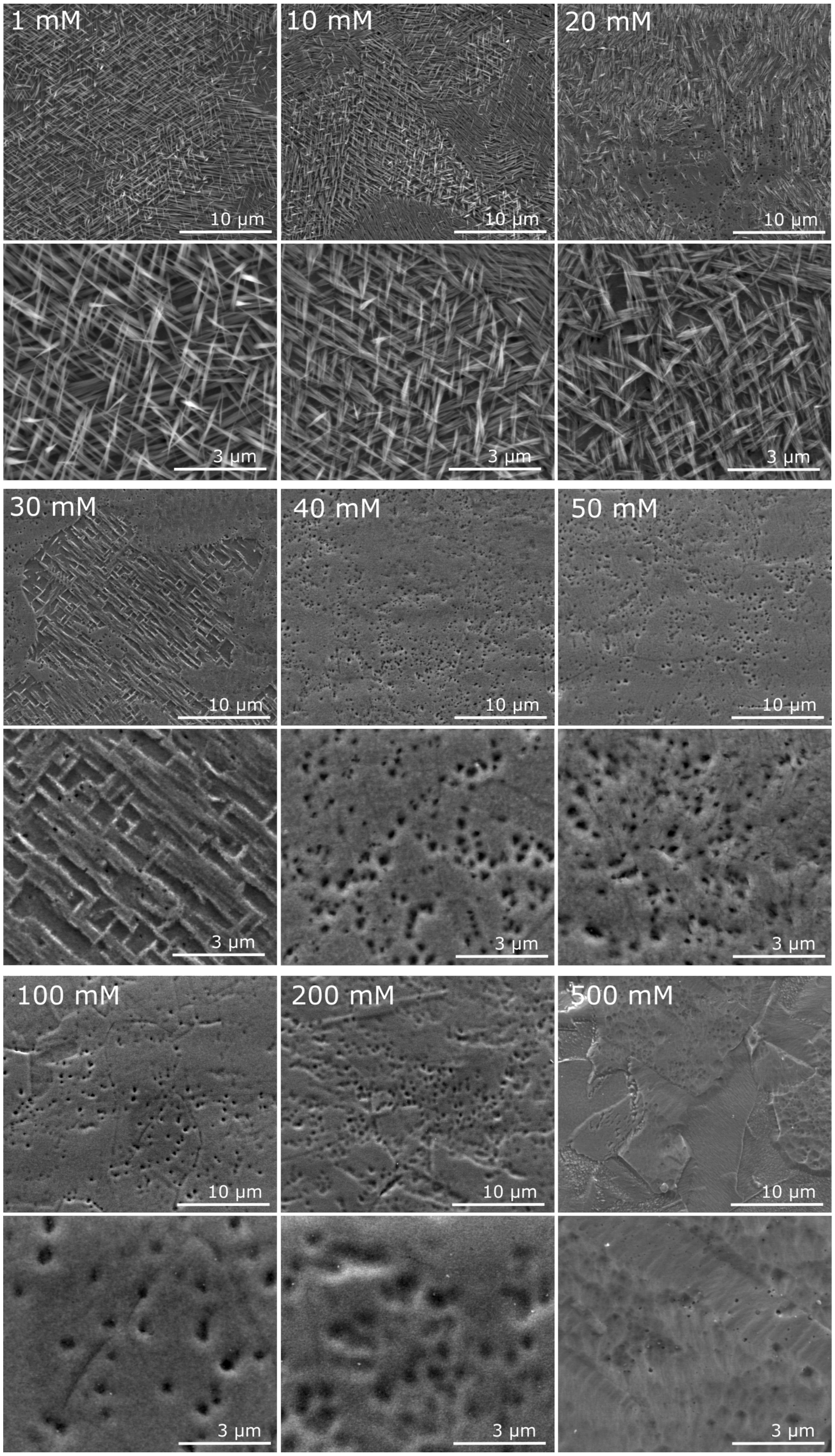

Figure S1: SEM images at two magnifications of the copper surface anodized in 1 M NaOH and EDTA at various concentrations.

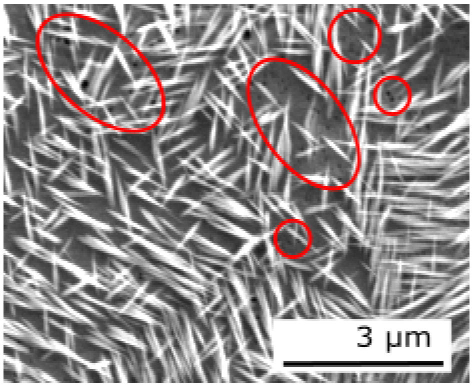

Figure S2: SEM image of copper anodized in 1 M NaOH and 10 mM EDTA for 5 s. Specific area of the surface with underlying pores is highlighted with red circles.

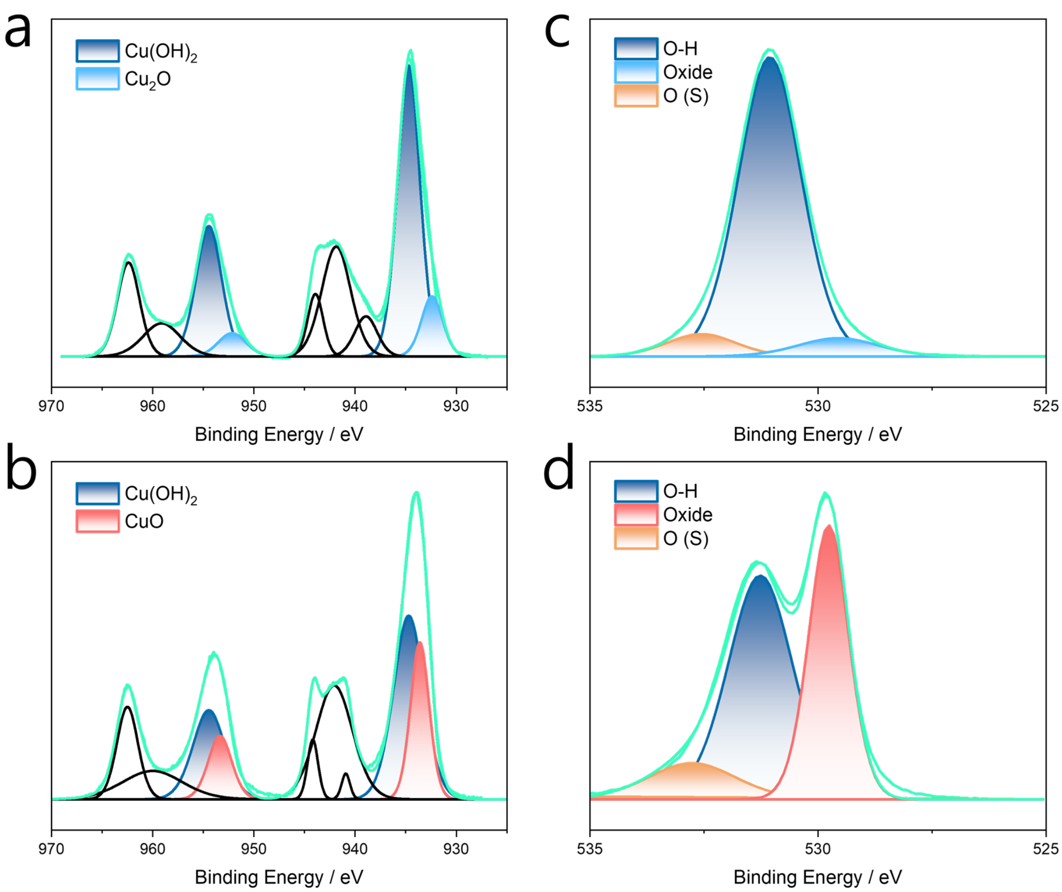

Figure S3: High-resolution XPS spectra of (a,b) Cu $2p$ and (c,d) O $1s$ regions for copper anodized in 1 M NaOH with EDTA at a concentration of (a,c) 0 and (b,d) 100 mM.

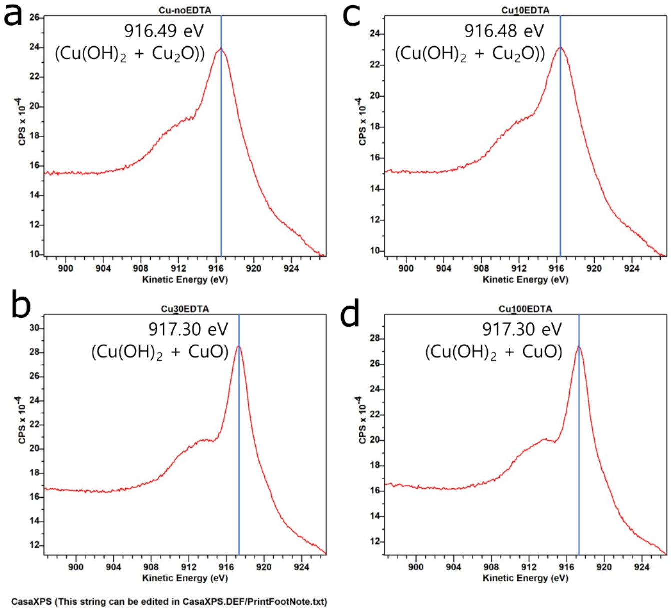

Figure S4: Auger spectra for copper anodized in 1 M NaOH with EDTA at a concentration of (a) 0, (b) 30, (c) 10, and (d) 100 mM.

Table S1: XPS fitting results of anodized copper under different EDTA concentrations.

EDTA Concentration (mM)	Measurement Region	Chemical State	Binding Energy (eV)		Peak 1 Area Ratio (%)
			Peak 1	Peak 2	
0	Cu 2p	Cu_2O	932.4	952.2	16.25
		$Cu(OH)_2$	934.7	954.5	83.75
	O 1s	Oxide	529.6	-	6.76
		O–H	531.1	-	86.12
		O (S)	532.6	-	7.13
10	Cu 2p	Cu_2O	932.4	952.2	15.57
		$Cu(OH)_2$	934.7	954.5	84.43
	O 1s	Oxide	529.6	-	9.03
		O–H	531.1	-	82.28
		O (S)	532.6	-	8.70
30	Cu 2p	CuO	933.6	953.4	34.43
		$Cu(OH)_2$	934.7	954.5	65.57
	O 1s	Oxide	529.8	-	39.00
		O–H	531.3	-	50.19
		O (S)	532.8	-	10.81
100	Cu 2p	CuO	933.6	953.4	34.36
		$Cu(OH)_2$	934.7	954.5	65.64
	O 1s	Oxide	529.8	-	38.24
		O–H	531.3	-	50.61
		O (S)	532.8	-	11.15
